# PROTAC-Based Antivirals for Respiratory Viruses: A Novel Approach for Targeted Therapy and Vaccine Development

**DOI:** 10.3390/microorganisms13071557

**Published:** 2025-07-02

**Authors:** Amith Anugu, Pankaj Singh, Dharambir Kashyap, Jillwin Joseph, Sheetal Naik, Subhabrata Sarkar, Kamran Zaman, Manpreet Dhaliwal, Shubham Nagar, Tanishq Gupta, Prasanna Honnavar

**Affiliations:** 1 Basic Medical Science, American University of Antigua College of Medicine, St. Johns 1543, Antigua and Barbuda; amitha@auamed.net (A.A.); shubhamn@auamed.net (S.N.); tanishqg@auamed.net (T.G.); 2 ICMR-National Institute for One Health, Nagpur, Maharashtra 440006, India; rawat.pankaj899@gmail.com; 3 Brown Center for Immunotherapy, Melvin and Bren Simon Comprehensive Cancer Center, Division of Hematology and Oncology, School of Medicine, Indiana University, Indianapolis, IN 46202, USA; dbir@iu.edu; 4 Department of Microbiology and Immunology, American University of Antigua College of Medicine, St. Johns 1543, Antigua and Barbuda; jjillwin@auamed.net; 5 Department of Physiology, American University of Antigua College of Medicine, St. Johns 1543, Antigua and Barbuda; snaik@auamed.net; 6 Department of Virology, Postgraduate Institute of Medical Education and Research, Chandigarh 160012, India; subhabrata5426@gmail.com; 7 Department of Microbiology and Molecular Biology, ICMR-National Institute of Traditional Medicine, Belagavi 590010, India; kamran3zaman@gmail.com; 8 Allergy and Immunology Unit, Department of Pediatrics, Advanced Pediatrics Centre, Postgraduate Institute of Medical Education and Research, Chandigarh 160012, India; manpreet326@gmail.com

**Keywords:** PROTAC, targeted protein degradation, antiviral, vaccine, ubiquitin–proteasome

## Abstract

The global burden of respiratory viral infections is notable, which is attributed to their higher transmissibility compared to other viral diseases. Respiratory viruses are seen to have evolved resistance to available treatment options. Although vaccines and antiviral drugs control some respiratory viruses, this control is limited due to unexpected events, such as mutations and the development of antiviral resistance. The technology of proteolysis-targeting chimeras (PROTACs) has been emerging as a novel technology in viral therapeutics. These are small molecules that can selectively degrade target proteins via the ubiquitin–proteasome pathway. PROTACs as a therapy were initially developed against cancer, but they have recently shown promising results in their antiviral mechanisms by targeting viral and/or host proteins involved in the pathogenesis of viral infections. In this review, we elaborate on the antiviral potential of PROTACs as therapeutic agents and their potential as vaccine components against important respiratory viral pathogens, including influenza viruses, coronaviruses (SARS-CoV-2), and respiratory syncytial virus. Advanced applications of PROTAC antiviral strategies, such as hemagglutinin and neuraminidase degraders for influenza and spike proteins of SARS-CoV-2, are detailed in this review. Additionally, the role of PROTACs in targeting cellular mechanisms within the host, thereby preventing viral pathogenesis and eliciting an antiviral effect, is discussed. The potential of PROTACs as vaccines, utilizing proteasome-based virus attenuation to achieve a robust protective immune response, while ensuring safety and enhancing efficient production, is also presented. With the promises exhibited by PROTACs, this technology faces significant challenges, including the emergence of novel viral strains, tissue-specific expression of E3 ligases, and pharmacokinetic constraints. With advanced computational design in molecular platforms, PROTAC-based antiviral development offers an alternative, transformative path in tackling respiratory viruses.

## 1. Introduction

Globally, respiratory viruses, including the influenza A and B viruses, respiratory syncytial virus (RSV), rhinovirus, adenovirus, parainfluenza virus, metapneumoviruses, bocavirus, and coronaviruses, are the most typical causes of respiratory infection in immunocompetent patients. In contrast, cytomegalovirus (CMV), herpes simplex virus (HSV), or varicella-zoster virus (VZV) can cause disease in immunocompromised patients [1]. Over the last few decades, there has been an increase in the incidence of respiratory virus outbreaks/epidemics/pandemics worldwide, such as the Nipah virus (1998), severe acute respiratory coronavirus 1 (SARS-CoV-1) (2003), Middle East respiratory syndrome coronavirus (MERS-CoV) (2012), severe acute respiratory syndrome coronavirus 2 (SARS-CoV-2) (2019), bird flu, etc. [2,3,4,5]. The high mortality and morbidity associated with these respiratory virus infections highlight the critical need for effective therapeutic interventions.

Traditionally, the prevention and control of respiratory viral infections rely on vaccines and antivirals. Effective vaccines or antiviral drugs are available for only a few respiratory viruses. High mutation rates of viruses such as the influenza virus and SARS-CoV-2 hinder the efficacy of available vaccines. However, the effectiveness of antiviral drugs, like neuraminidase (NA) inhibitors (oseltamivir) and M2 ion channel blockers (amantadine), is limited by the emergence of drug-resistant viral strains and adverse side effects [6]. The dynamics of respiratory virus evolution emphasize the critical need for advanced novel technologies to develop effective vaccines and antiviral drugs. Over the past few years, targeted protein degradation (TPD) has emerged as a promising technology in the development of medical countermeasures (MCMs), such as proteolysis-targeting chimeras (PROTACs). Thus, we evaluate the advancement and application of PROTAC technology in the field of virology. This review summarizes the advancements in PROTAC-based antivirals and PROTAC vaccines, the challenges, and future prospects.

## 2. PROTAC

**Figure 1:**
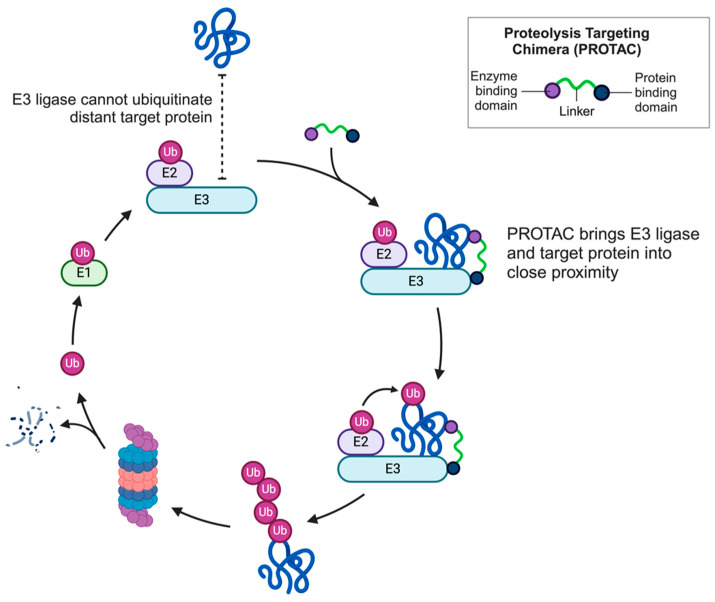
PROTAC’s mechanism of action: PROTAC is a small molecule composed of two active domains and a linker. One of the domains binds to the target protein. In contrast, the other binds to the E3 ligase enzyme, bringing the two proteins into proximity, which causes the ubiquitination of the target protein, followed by its degradation through the proteasomal pathway. This can enable us to eliminate target proteins selectively (Ub: ubiquitin).

PROTAC is an emerging technology in targeted protein degradation (TPD) that aims to develop therapeutic agents. PROTAC molecules are composed of two linked domains: one domain binds to the protein of interest (POI), and the other binds to the E3 ubiquitin ligase. The formation of POI–PROTAC–E3 ternary complexes facilitates the ubiquitylation of the POI, leading to its subsequent degradation by the proteasome (Figure 1). Since PROTACs remain intact during this process, a single PROTAC molecule can instigate the ubiquitination and degradation of multiple POI equivalents [7]. Although PROTAC technology is emerging as a viable therapeutic strategy in cancer research and treatment, its application in antimicrobial treatment remains largely unexplored. PROTACs offer numerous advantages over traditional protein inhibitors (drugs), including effectiveness at low concentrations, rapid action, prolonged biological effects, specificity, lack of toxicity, robust immunogenic response, capacity to degrade undruggable targets, and effectiveness against resistant strains. Because of these advantages, many pharmaceutical companies are investing in PROTAC therapy for the treatment of both infectious and non-infectious diseases [8,9]. PROTAC is a progressing technology with a vast scope in protein degradation-based therapies for a wide range of pathological conditions.

### 2.1. PROTAC Design and Synthesis

Each PROTAC is composed of three main parts: a warhead (a ligand that attaches to the POI), an anchor (a ligand that attaches to an E3 ubiquitin ligase), and a linker that connects the two. This setup enables the PROTACs to degrade both druggable and undruggable targets, paving the way for novel antimicrobial developments.

#### 2.1.1. Warhead

The first step in PROTAC design involves identification of the POI and the POI ligands. The PROTAC event-driven catalytic mechanism of action does not require strong binding with the POI, unlike conventional drugs. The PROTAC’s efficacy depends on the POI-PROTAC-E3 ligase ternary complex formation.

The PROTAC has ability to target approximately 80% human proteins, which includes transcription factors (c-Myc, STAT3), RNA-binding proteins, epigenetic targets, kinases (Bruton Tyrosinase Kinase), receptors (AR, ER, RAR, PR, EPGF), scaffold proteins (beta-catenin, KSR1/2, p62/SQSTM1, NEMO/IKKγ, Axin1/2), and mutant proteins, eliminating the limitations of traditional drugs [10]. The strategy used for targeting POIs includes inhibitor-based warheads (indomethacin-based PROTACs and cyclin-dependent kinase-based PROTACs) and antimicrobial-based warheads (oseltamivir-based PROTACs and telaprevir-based PROTACs) [11,12,13].

#### 2.1.2. Linker

**Figure 2:**
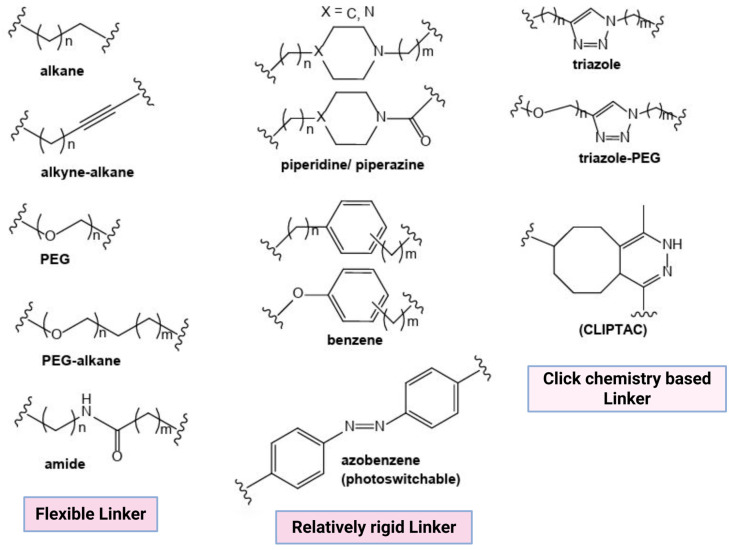
Structure of some common PROTAC linkers.

Linkers are the connecting link between the anchor (E3 ligase-binding domain) and a “warhead” (POI-binding domain). PROTAC selectivity, solubility, stability, flexibility, and degradation efficacy depend on the linker length, type, and composition [14]. Thus, in order to design PROTACs with desired characteristics, the linker properties are modified. The most commonly used PROTAC linkers include polyethylene glycol, triazole, alkyl chains, piperazine, and amide bonds (Figure 2).

- Linker length: Linker length determines the PROTAC target protein degradation efficacy; long linkers may hinder POI ubiquitination, whereas short linkers minimize the chances of ternary complex (TC) formation and result in a hook effect, i.e., binary complex formation (PROTAC-E3 ligase complex) [15].
- Flexibility: Flexible linkers, such as alkyl chains and PEG, increase PROTAC flexibility, whereas linkers like alkynes, piperazine, and triazole increase PROTAC rigidity. Alkyl, PEG, and extended PEG are the most commonly used linkers because of their easily controlled flexibility [15].
- Chemical composition: PROTACs with a high molecular weight result in poor pharmacokinetic–pharmacodynamic (PK/PD) properties. Multiple warhead–linker–anchor combinations can be screened to select a PROTAC with the desired PK/PD properties. The hydrophilic linkers (PEG) increase the bioavailability and solubility of PROTAC molecules. On the contrary, hydrophobic linkers improve the PROTAC’s cellular permeability [14,15].

#### 2.1.3. Anchor

**Figure 3:**
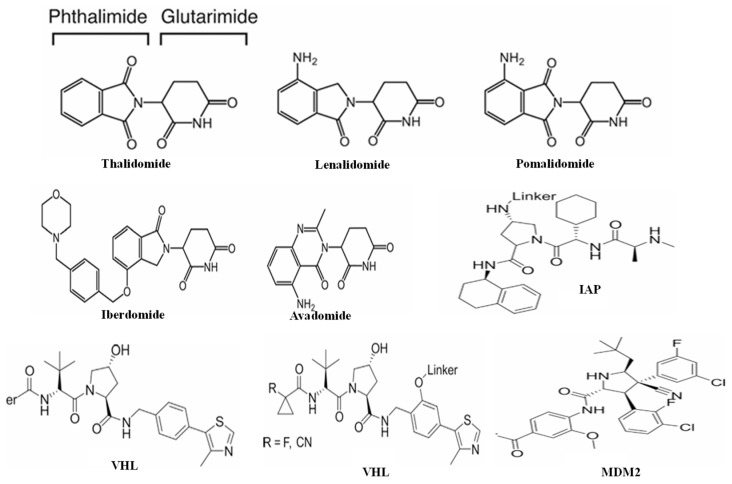
**E3 ligases commonly used in PROTAC technology** (VHL: Von Hippel–Lindau; MDM2: mouse double minute 2 homologue; IAP: inhibitor of apoptosis protein).

Despite the existence of over 600 E3 ligases, only a few E3 ligases are explored in PROTAC technology, which includes Von Hippel–Lindau (VHL) E3 ligases, mouse double minute 2 homologue (MDM2) E3 ligases, cerebelon (CRBN) E3 ligases, inhibitor of apoptosis protein (IAP) E3 ligases, aryl hydrocarbon receptor (AhR), DDB1- and CUL4-Associated Factor 15 (DCAF15), RING finger 14 (RNF14), DCAF16, and Kelch-like ECH-associated protein 1 (KEAP1) (Figure 3) [14]. CRBN and VHL are extensively used in PROTAC technology because of their ligand availability, proven efficacy, low toxicity, linker compatibility, warhead compatibility, structure availability, and widespread tissue expression [14].

#### 2.1.4. PROTAC Synthesis

PROTAC synthesis is a time-consuming, complex process. Various approaches have been used for PROTAC synthesis, including the coupling of one ligand bearing the linker with another ligand using activated esters, alkylation reactions, Staudinger ligation chemistry, multicomponent synthesis, solid-phase synthesis, click chemistry, the rapid synthesis of PROTACs (Rapid-TAC) platform, and the modular synthetic platform [16,17,18,19]. The in-silico tools available for designing PROTACs include DeLinker, SyntaLinker, PRossettaC, and commercial tools such as ICM modelling (ICM-Pro v.3.9) and molecular operating environment (MOE 2024.06) [20,21,22].

## 3. PROTAC-Based Antivirals

PROTAC is a TPD technology that requires the identification of the protein of interest (POI) to develop effective antiviral therapeutics. Viruses are infectious agents composed of proteins and nucleic acids (DNA or RNA), often surrounded by a lipid membrane in certain species, known as an envelope. The virus envelope is acquired from the host cell membrane and contains both virus-derived and host-derived proteins, which play a critical role in virus attachment and entry [23]. All viruses complete their life cycle in living cells, which involves six crucial steps: attachment, penetration, uncoating, replication, assembly, and release [24]. During these stages, the virus utilizes multiple proteins (both viral and host proteins) for viral entry (spike, hemagglutinin, F protein, ACE2), viral genome replication (RNA-dependent RNA polymerase), virus particle assembly, and virus particle release (neuraminidase), which can differ among various viruses [24,25,26]. PROTACs can target both viral and host proteins to inhibit viral replication. Therefore, a comprehensive understanding of the viral life cycle, facilitated by advanced technology, is crucial for the identification and selection of target proteins for PROTAC-based antiviral therapies.

**Figure 4:**
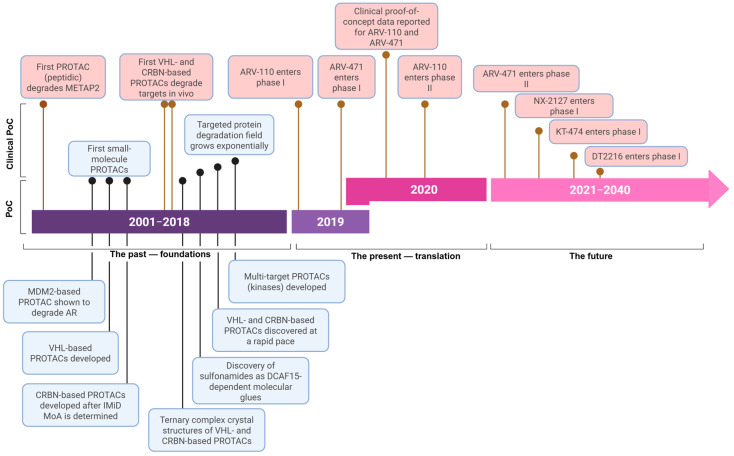
Timeline for PROTAC discoveries.

The era of TPD began with the introduction of PROTACs in 2001, marking the initial demonstration that the ubiquitin–proteasome system (UPS) could be intentionally exploited for the degradation of target proteins [27]. Since then, the field has significantly expanded, evolving from protein-based PROTACs to click-release PROTACs, folate-targeting PROTACs, photo-switchable PROTACs, radiation-responsive Protac, antibody–ProtAC conjugates, aptamer–ProtAC conjugates, and nano-ProtAC polymers [28]. The foundational era of TPD reached its peak with the entry of the first PROTAC AR degraders in a clinical trial (Figure 4) [29]. The PROTAC era is under the the translational phase, with numerous molecules designed for TPD progressing toward clinical use, aiming to significantly benefit patients. Table 1 listed the PROTACs-based antivirals.

**Figure 5:**
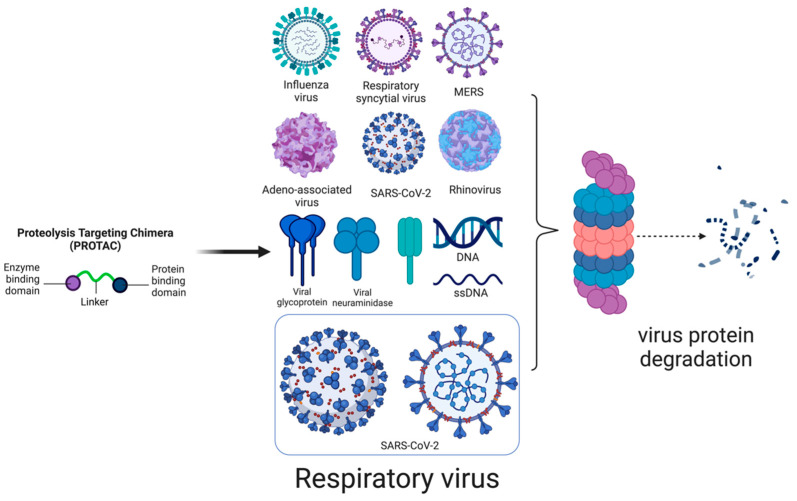
Respiratory viruses: proteins targeted for PROTAC-based therapy.

The common viral families that cause respiratory diseases are Orthomyxoviridae (influenza virus), Paramyxoviridae (parainfluenza viruses 1–4), Pneumoviridae (RSV and metapneumovirus), Picornaviridae (rhinovirus), Coronaviridae (MERS-CoV, SARS-CoV-1, and SARS-CoV-2), and Adenoviridae (adenovirus) [30]. In these viruses, antiviral drugs may target either viral proteins, such as structural proteins (capsid proteins, envelope proteins, and matrix proteins), or non-structural proteins (polymerases, proteases, viral ribonucleoproteins, and accessory proteins) (Figure 5). PROTACs targeting viral proteins are safe for hosts because they focus on viral-specific proteins with no human homologs.

### 3.1. Protac-Based Antivirals for Influenza Virus

#### 3.1.1. PROTAC-Based Antivirals Targeting Hemagglutinin

**Figure 6:**
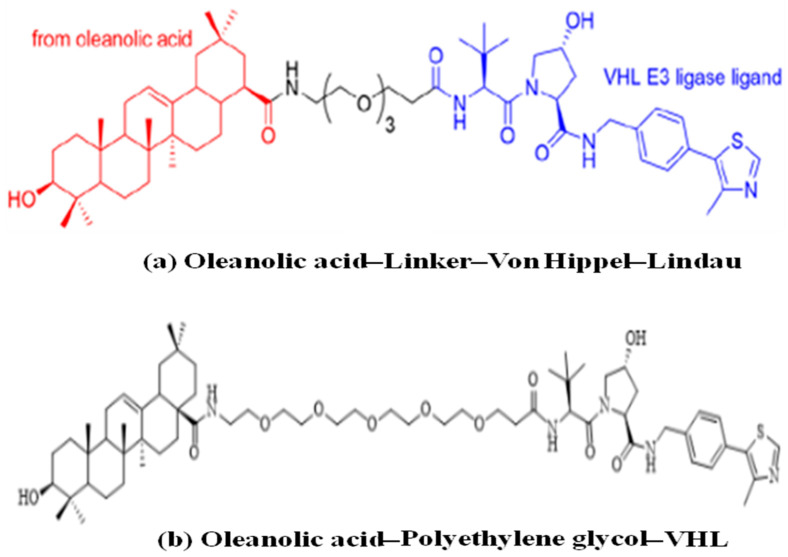
Illustrating the pentacyclic triterpenoid-based PROTACs against the influenza A virus targeting hemagglutinin. (**a**) Oleanolic acid–linker–Von Hippel–Lindau. (**b**) Oleanolic acid–polyethylene glycol–VHL.

Hemagglutinin (HA) and neuraminidase (NA) are the surface glycoproteins of the influenza virus that play crucial roles in infection. HA is the most abundant glycoprotein on the surface of the influenza virus. It facilitates viral attachment and fusion by interacting with sialic acid on the host cell surface and initiates virus entry via endocytosis. The immune response to influenza infection, antivirals, or vaccination primarily focuses on the HA protein. The PROTACs targeting hemagglutinin include oleanolic acid-based PROTACs. Li et al. (2022) [31] developed these degraders for influenza HA using protein degradation technology. These novel pentacyclic triterpenoid-based PROTACs enhance the degradation of the HA target in a ubiquitin–proteasome-dependent manner and exhibit broad-spectrum activity against the influenza A virus ( Figure 6). Furthermore, in vivo studies using animal models also demonstrated strong antiviral activity, suggesting that oleanolic acid-based PROTACs could serve as potential antiviral agents for treating influenza [31,32].

#### 3.1.2. PROTAC-Based Antiviral Targeting Neuraminidase

NA, a surface glycoprotein similar to HA, plays a critical role in the interaction of viruses with sialic acid on the host cell surface. Without NA activity, the progeny influenza virus remains aggregated and cannot be released to initiate a new round of infection. As a result, the virus is not amplified, and the infection does not progress further [33]. NA-specific antibodies are protective and effective in reducing virus shedding and the severity of infection. NA has been recognized as a primary drug target for the prophylaxis and treatment of influenza infections. Neuraminidase inhibitors (NAIs) such as oseltamivir (Tamiflu), zanamivir, and peramivir are the most commonly prescribed and utilized drugs for the prophylaxis and treatment of human influenza [34]. These drugs inhibit the synthesis of virus particles by blocking NA activity. Despite the significant success in developing influenza neuraminidase inhibitors (NAIs), an ongoing need remains to synthesize new antivirals due to the emergence of drug-resistant strains. Frequent use of oseltamivir has resulted in drug-resistant mutants [6].

**Figure 7:**
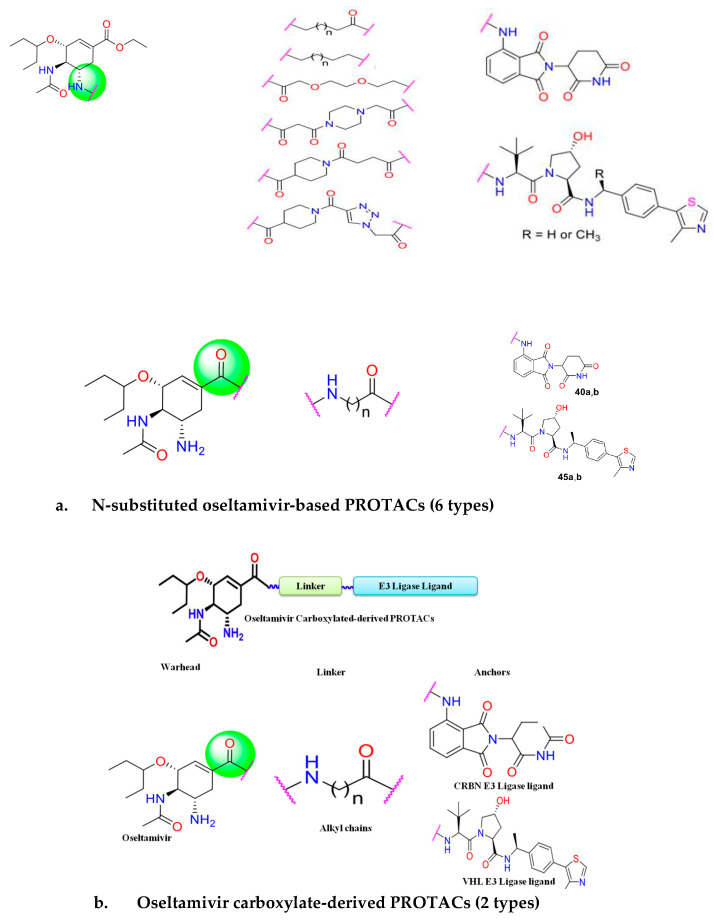
Illustrating the oseltamivir-based PROTACs: (**a**) N-substituted oseltamivir PROTACs (6 types): series-I, multiple PROTACs designed using different combinations of linkers and E3 ligase ligands; (**b**) oseltamivir carboxylate-derived PROTACs (2 types): series-II, multiple PROTACs designed using different combinations of linkers and E3 ligase ligands.

Recently, Xu et al. (2022) [12] developed PROTAC degraders that target the influenza NA protein, using an oseltamivir scaffold to tackle the issue of drug resistance. The oseltamivir-based PROTACs incorporate the E3 ligase ligands VHL or CRBN along with various linkers: N-substituted oseltamivir PROTAC (series-I) and N-carboxylated oseltamivir PROTAC (series-II) (Figure 7). These compounds demonstrate high potency against H1N1 proliferation in vitro. Additionally, the degraders show strong antiviral activity against both wild-type (H1N1) and oseltamivir-resistant strain of influenza virus (H274Y) [12].

#### 3.1.3. Others

**Figure 8:**
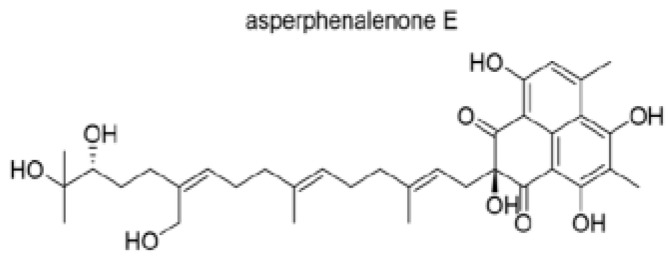
Structure of asperphenalenone E (APL-16-5).

Asperphenalenone E (APL-16-5) is a natural compound isolated from Aspergillus sp. CPCC 40073512 (Figure 8). This compound exhibits potent anti-influenza A virus activity in both in vitro and in vivo models. Like PROTAC, APL-16-5 binds to both the E3 ligase TRIM25 and the influenza A virus PA subunit, leading to the proteasomal degradation of the PA subunit and inhibition of viral replication (Figure 8).

### 3.2. Protac-Based Antiviral for SARS-CoV-2

The SARS-CoV-2 genome contains multiple (13 to 15) open reading frames (ORFs) that encode 27 viral proteins (structural and non-structural). ORF1ab (ORFa/b) by frameshift mutation encodes 16 non-structural proteins (NSPs) by the protease activity of two cysteine proteases: PLpro (nsp3) and Mpro (nsp5). The significant SARS-CoV-2 antiviral target proteins are spike, envelope, membrane, RNA-dependent RNA polymerase (RdRP), and nucleocapsid proteins [35]. Among these proteins, the antiviral agents such as remdesivir target RdRP and Nirmatrelvir/ritonavir to the SARS-CoV-2 protease [36].

#### SARS-CoV-2 Computational Platform for PROTAC-Based Antiviral

Chatterjee et al. (2020) [37] developed a computational platform for designing ACE2-derived peptides that target the receptor-binding domain (RBD) of the SARS-CoV-2 spike protein and recruit E3 ubiquitin ligase for subsequent proteasomal degradation. The engineered peptide fusions (PROTACs) demonstrate significant anti-SARS-CoV-2 activity in a cell line model, prompting further research into therapeutic development [37]. This computational PROTAC design pipeline can also be applied to other viruses. Similarly, Shaheer et al. (2021) [38] established a computational platform for PROTAC design, utilizing protein-protein docking to identify complementary binding sites between cereblon E3 ligase and Mpro of SARS-CoV-2, as well as to estimate potential linker lengths. Furthermore, molecular dynamics simulations of PROTAC reveal strong interactions and the potential for proteasomal degradation of the target protein [38].

## 4. PROTAC Targeting Host Proteins

Antiviral agents that target viral proteins encounter several limitations, including narrow-spectrum activity and the emergence of antiviral resistance [39]. Consequently, the effectiveness of current antivirals diminishes or vanishes as new viruses emerge and pathogens evolve. Antivirals that target human proteins may help address this challenge by enabling the development of pan-antiviral agents that are less likely to encounter microbial resistance [40]. Therefore, host-targeted antivirals (HTAs) are a promising approach for emerging, re-emerging, and novel viral infections.

### 4.1. Human Prostaglandin E Synthase Type 2 (PGES-2)-Based PROTAC

**Figure 9:**
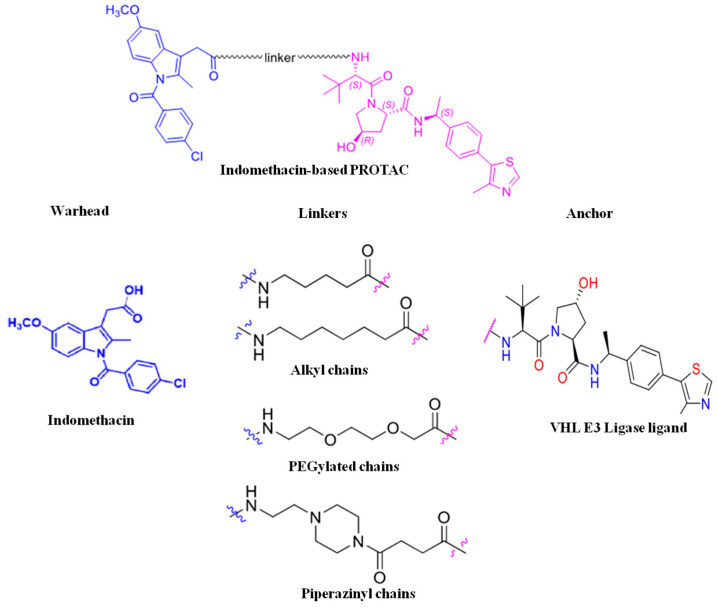
Indomethacin-based PROTAC: structure of INM-based PROTACs.

Targeting host proteins to prevent viral replication, Desantis et al. [11] developed Indomethacin (INM)-based PROTACs that exhibit anti-SARS-CoV-2 activity (Figure 9). These PROTACs were created by linking INM with the Von Hippel–Lindau (VHL) E3 ligase ligand using aliphatic and polyethylene glycol (PEG) linkers. Biological evaluations of the INM-based PROTACs demonstrated broad-spectrum inhibitory activity against both pandemic and epidemic coronaviruses of the Coronaviridae family [11]. Molecular modeling studies also suggest that PGES-2 could serve as a potential target for INM-based antiviral PROTACs, thereby paving the way for developing host-directed anti-CoV strategies [11].

### 4.2. Cyclin Dependent Kinase (CDK)-Based PROTAC

**Figure 10:**
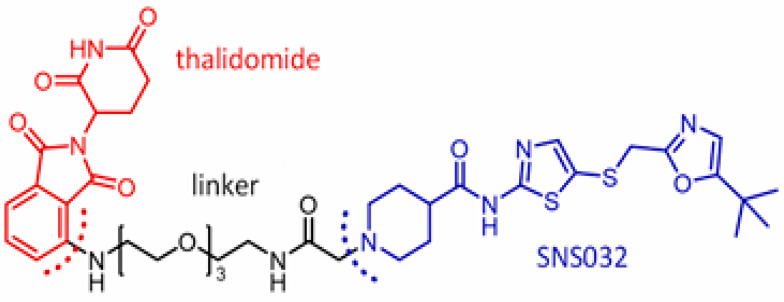
Illustrating cyclin-dependent kinase-based PROTAC 9 (THAL-SNS032, SN032–linkers–thalidomide (**Cereblon**)): protein of interest = cyclin-dependent kinase.

CDKs are crucial in regulating the host cell cycle and transcription processes [41]. Viruses (RNA and DNA) alter CDK expression to influence cellular functions as viral replication depends on the host cell machinery. For example, the influenza virus and SARS-CoV-2 disrupt the cell cycle at the G0/G1 and S/G2 phases, respectively, to establish a favorable environment for viral replication [42,43]. Pharmacological CDK inhibitors (PCDKis), which were developed and approved for cancer therapy, can be repurposed for antiviral treatment. These PCDKis-based PROTACs represent promising candidates for broad-spectrum antiviral agents [44]. THAL-SNS032, a CDK9-directed PROTAC, exhibits broad-spectrum antiviral activity in an in vitro model, inhibiting the replication of human cytomegalovirus (HCMV), SARS-CoV-2, and murine cytomegalovirus (Figure 10).

### 4.3. PROTAC-Based Antiviral for Virus-Induced Cytokine Storm

**Figure 11:**
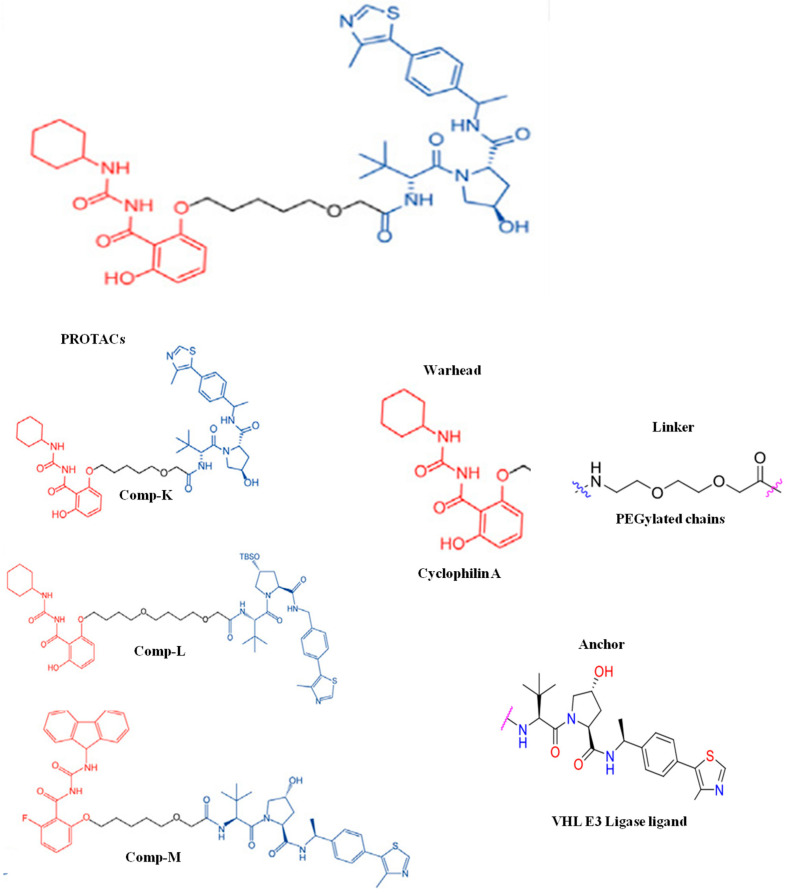
Cyp A-based PROTACs.

Respiratory viruses trigger cytokine storms in patients, leading to uncontrolled inflammation resulting in acute respiratory distress syndrome (ARDS) and multi-organ failure [45]. Currently, the therapy for controlling cytokine storms includes steroids, cytokine inhibitors, and immunoglobulin. A study by Heqiao Li et al. (2023) reported the high efficacy of a cyclophilin A (CypA)-targeting PROTAC in controlling cytokine storms (Figure 11). In brief, the author designed the PROTAC for CypA using molecular docking. The PROTAC-mediated depletion of Cyp A reduced the secretion of inflammatory cytokines and cellular injury in both the cell line and the mouse model [46].

## 5. Protac Vaccine

Vaccination remains one of the most effective and straightforward tools for preventing and controlling viral infections, including respiratory viruses. Vaccination strategies and platforms encompass vaccines derived from attenuated pathogens, inactivated pathogens, and subunits (such as protein subunits, virus-like particles, nucleic acid, and mRNA) [47]. Within these strategies, live-attenuated vaccines (LAVs) are the most effective interventions against viral infections as they induce humoral, cellular, and herd immunity. Currently, several approaches for producing LAVs are being proposed, including codon-deoptimization vaccines, cold-adapted live-attenuated influenza vaccines (CAIVs), premature termination codon (PTC)-harboring viruses, viral-protein-altered viruses, and hyper-interferon-sensitive viruses [48,49,50,51]. However, current attenuation strategies encounter unpredictable attenuation, compromised vaccine safety, suboptimal immunogenicity, decreased vaccine efficacy, and complicated production processes [52]. Moreover, virus evolution poses an additional challenge to vaccine efficacy. Therefore, there is an urgent need for novel technologies such as PROTACs to develop safer and more effective live vaccines. Unlike traditional vaccine production methodologies, PROTACs utilize the ubiquitin–proteasome pathway to induce an efficient immune response. PROTAC technology surpasses other attenuation methodologies in terms of enhanced safety, efficacy, robust immune response, and reduced production costs [53]. Thus, PROTAC technology presents a promising approach for generating more effective and safer viral vaccines.

Recently, Si et al. described a novel PROTAC-based vaccine technology. They utilized PROTAC technology to attenuate the influenza virus by leveraging the host proteasomal degradation pathway. The attenuated strain of the influenza virus (PROTAC virus) was created by attaching a proteasome-targeting domain (PTD) to viral structural or non-structural proteins. The PROTAC viruses were generated by linking the PTD to target proteins. To regulate the replication of the PROTAC virus in cell lines, the PTD peptide was connected to the target protein through a tobacco etch virus (TEV) protease-sensitive site (ENLYFQG). The TEV protease-expressing cell lines prevent the proteolysis of the PTD-tagged target proteins. Consequently, the PROTAC virus replicates in cell lines that stably express TEV protease, producing the PROTAC virus strains for vaccine production [54].

The effectiveness of the PROTAC vaccine depends on the host ubiquitin–proteasome system. Si et al. evaluated the PROTAC vaccine efficacy for the influenza A virus using conventional Madin-Darby canine kidney 2 (MDCK2) and TEV-expressing MDCK2 (MDCK-TEVp) cell lines. The PTD tagging led to the efficient degradation of viral proteins in MDCK2 cells. Among the eight proteins, M1-PTD displayed the highest antiviral activity in cell lines and animal models [53,54]. Furthermore, the PROTAC vaccine produced a strong humoral and cellular immune response compared to the inactivated influenza vaccine (IIV) and the CAIV [54,55].

**Figure 12:**
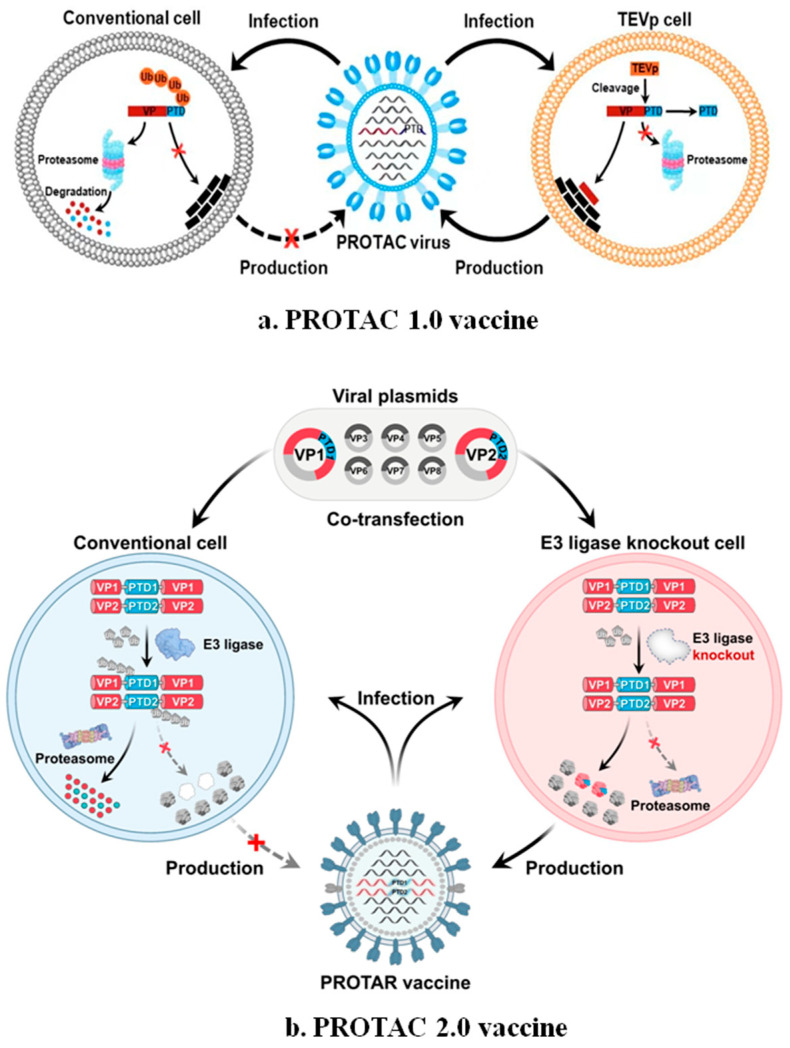
Illustration of the generation of PROTOC 1.0 and 2.0 vaccines .

Proteolysis-targeting chimera (PROTAC) 2.0, the next generation of the PROTAC vaccine approach, includes the insertion of PTD in multiple sites within target proteins, including the N-terminus, internal region, and C-terminus (Figure 12). The PROTAR 2.0 viruses were efficiently replicated in E3 ubiquitin ligase-deficient cell lines. Still, no replication was observed in conventional cell lines. In animal models, PROTAR 2.0 vaccines induced a broad humoral and cellular immune response [56].

## 6. Challenges

PROTAC technology employs the host protein degradation system. The target proteins (POIs) for PROTACs can include druggable enzymes and proteases, as well as challenging targets such as DNA-binding proteins, signal transduction proteins, and transcriptional enhancers [8]. As a result, antiviral PROTACs show promise in targeting complex proteins involved in virus replication and suppressing the host immune response. Furthermore, PROTACs can also target virus mutants, offering a promising approach to tackling antiviral resistance.

The PROTAC-based therapeutics for respiratory viruses may encounter several challenges, including virus evolution (the emergence of novel viruses, the evolution of known viruses such as IAV, SARS-CoV-2 etc), limited understanding of viral pathogenesis, tissue-specific expression of E3 ligase, a small number of antiviral agents with specific targets, PROTACs poor pharmacokinetic (PK) and pharmacodynamic (PD) properties, an underdeveloped clinical evaluation system for PROTACs, and a complex, time-consuming process of PROTAC synthesis and screening [14,16,17,18,19,24].

## 7. Conclusions and Perspectives

Antiviral drug discovery is a challenging, time-intensive, and expensive process with a very low success rate. Additionally, existing antiviral agents are becoming less effective due to the emergence of drug resistance. Although PROTAC-based antiviral strategies are still in their early phases, their broad-spectrum activity against both wild-type and mutant strains presents advantages over traditional inhibitors. However, their success depends on expanding the repertoire of POI, E3 ligases, virus tissue tropism, viral pathogenesis, and virus evolution. Therefore, the rational design of an antiviral PROTAC molecule must consider the cellular proteasomal degradation system and viral pathogenesis. The PROTAC design process is complex; previously published computational methods or pipelines (in silico tools) can be utilized to expedite the development of PROTAC-based antiviral agents for other viruses. Furthermore, advanced technologies such as artificial intelligence and established PROTAC-related databases (including PROTAC-DB 3.0, DiffPROTAcs, PROTACpedia, and the DeepPROTACs predictor) can aid in the rational design of PROTAC-based antiviral agents [57,58]. Advance PROTAC technology such as Click-formed PROTAC (CLIPTAC), nano-PROTAC, and antibody–PROTAC conjugates (Ab-PROTAC) will improve the PROTAC delivery and stability [59,60,61,62]. Furthermore, other biomolecule degradation technologies, including ribonuclease-targeting chimera (RIBOTAC), autophagy-targeting chimera (AUTAC), lysosome-targeting chimeras (LYTACs), and Autophagy-Tethering Compounds (ATTECs), are expected to play a significant role in the future development of antiviral agents [63,64,65,66].

## Figures and Tables

**Table 1:** PROTAC-based antivirals.

PROTAC-Based Antivirals	Structure
a. Telaprevir-based PROTACs (DGY-08-097) targeting HCV NS3/4A protease.	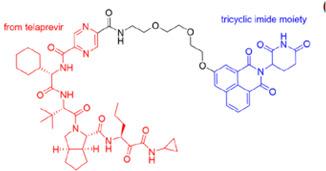
b. Indomethacin-based PROTACs targeting SARS-CoV-2.	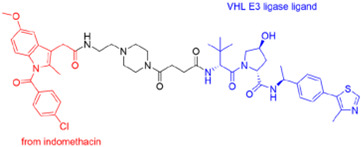
c. Oleanolic acid-based PROTACs targeting IAV Hemagglutinin protein.	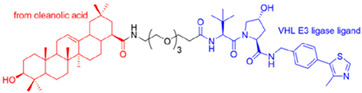
d. Oseltamivir-based PROTACs targeting IAV neuraminidase protein.	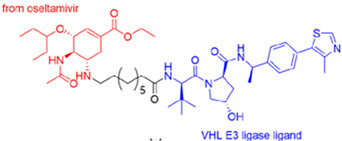
e. Cyclin-dependent kinase-based PROTACs (Thal-SNS032) for the inhibition of HCMV, SARS-CoV-2.	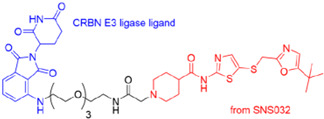
f. APL-16-5 (asperphenalenone E) for the inhibition of IAV.	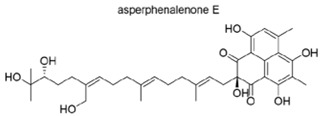
g. Macrocycle-based PROTACs targeting HCV and HIV-1.	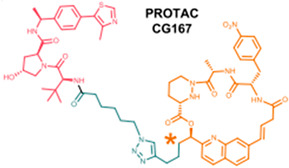

HCV: hepatitis C virus; IAV: influenza A virus; HCMV: human cytomegalovirus; SARS-CoV-2: severe acute respiratory syndrome coronavirus; HIV-1: human immunodeficiency virus.
